# Ocean warming and acidification modify top-down and bottom-up control in a tropical seagrass ecosystem

**DOI:** 10.1038/s41598-021-92989-0

**Published:** 2021-06-30

**Authors:** Vina Listiawati, Haruko Kurihara

**Affiliations:** 1 Faculty of Science, University of the Ryukyus, Okinawa, 903-0123 Japan; 2 Master of Marine Science, Universitas Diponegoro, Semarang, 50275 Indonesia; 3 Department of Biology Education, Universitas Muhammadiyah Surakarta, Surakarta, 57162 Indonesia

**Keywords:** Climate change, Ocean sciences

## Abstract

Seagrass ecosystem is one of the most productive ecosystems in coastal waters providing numerous ecological functions and supporting a large biodiversity. However, various anthropogenic stressors including climate change are impacting these vulnerable habitats. Here, we investigated the independent and combined effects of ocean warming and ocean acidification on plant–herbivore interactions in a tropical seagrass community. Direct and indirect effects of high temperature and high *p*CO_2_ on the physiology of the tropical seagrass *Thalassia hemprichii* and sea urchin *Tripneustes gratilla* were evaluated. Productivity of seagrass was found to increase under high *p*CO_2_, while sea urchin physiology including feeding rate decreased particularly under high temperature. The present study indicated that future climate change will affect the bottom-up and top-down balance, which potentially can modify the ecosystem functions and services of tropical seagrass ecosystems.

## Introduction

Seagrass ecosystems are classified as one of the most productive ecosystems in coastal waters^1^ and provide numerous ecological functions including regulation of the nutrient cycle^2^, carbon sink^3^, sediment stabilization^4^, and habitats and food provision for a wide range of marine organisms^5^. However, seagrass ecosystems are now threatened by a number of anthropogenic stressors, such as eutrophication, dredging and coastal development^6^. In addition to such local stressors, the ongoing global ocean environmental change caused by increasing atmospheric CO_2_ is expected to intensely affect seagrass ecosystems worldwide^7^. Therefore, information on how global climate change including ocean warming and acidification will affect seagrass meadows is critical to enable prediction and implementation of effective conservation strategies of seagrass ecosystems^8^.

Increase of seawater temperature is generally known to increase seagrass metabolism and productivity^9,10^. However, seagrass species are known to have a thermal tolerance window^11^, and temperature that exceed this window will negatively or potentially lethally affect them^12,13^. Meanwhile, ocean acidification has been suggested to benefit seagrasses and increase their productivity^14–16^ because photosynthesis of most seagrasses has been shown to be undersaturated at present seawater partial pressure of CO_2_ (*p*CO_2_)^17^. Therefore, although most studies have addressed the positive effects of ocean acidification on seagrasses, these benefits could be negated under the more realistic scenario of ocean acidification combined with an increase in temperature^18^.

What is less clearly established is how climate change will affect seagrass ecosystems through biological interactions such as between plant and herbivores^19^. For example, increase in temperature can increase the metabolism of herbivores in seagrass ecosystems such as keystone species of sea urchins, resulting in increased grazing pressure by the herbivores on the seagrass^20^. However, the grazing pressure can also be decreased due to ocean acidification through negative impacts on the herbivores^21,22^. Ocean acidification can also alter the C:N ratio and secondary metabolites such as phenolic and tannin of seagrass^18,23^ which may alter the plant’s resistance to herbivores. Therefore, it can be suggested that the combined effects of ocean warming and acidification will alter the top-down control of herbivores and the bottom-up control by the seagrass, which may result in a change in the equilibrium regulating seagrass ecosystems. However, to our knowledge, there are only two laboratory studies that have evaluated the effects of ocean acidification^19,24^ and one study^20^ evaluating the effect of ocean acidification and warming on interactions among seagrass and its consumers. Additionally, all studies are restricted to temperate species and there is no work addressing the effects of ocean warming and acidification on the tropical seagrass-herbivore interactions. Tropical seagrass species could be particularly sensitive to climate change, as most species may already be living at temperature close to their thermal limit^25^. Changes in seagrass abundances would have cascading effects over the entire ecosystem and therefore it is essential to evaluate the effects of climate change on tropical species interactions.

Here we investigate the independent and combined effects of ocean warming and acidification on both tropical seagrass species *Thalassia hemprichii* [(Ehrenberg) Ascherson, 1871] and sea urchin *Tripneustes gratilla* (Linnaeus, 1758) and their potential synergistic interactions. We designed a laboratory-based experiment in which seagrass and sea urchins were cultured under controlled high temperature (plus 3 °C than control) and high *p*CO_2_ (900–1000 µatm). We hypothesized that although the productivity of *T. hemprichii* will be enhanced by both high temperature and high *p*CO_2_ conditions, the feeding ability of *T. gratilla* will be enhanced by high temperature while it will be reduced by high *p*CO_2_ conditions. We first tested the direct effects of warming and acidification on the productivity and photo-physiological responses of the seagrass. Secondly, we examined the physiology including feeding, fecal production, respiration and ammonium (NH_4_^+^) excretion rates of sea urchins cultured under warming and/or acidification conditions and fed with two seagrass treatments; control seagrass which were cultured under the ambient control condition and experimental seagrass which were cultured under the same warming and/or acidification conditions as the sea urchins were cultured.

## Results

### Seagrass growth

**Figure 1:**
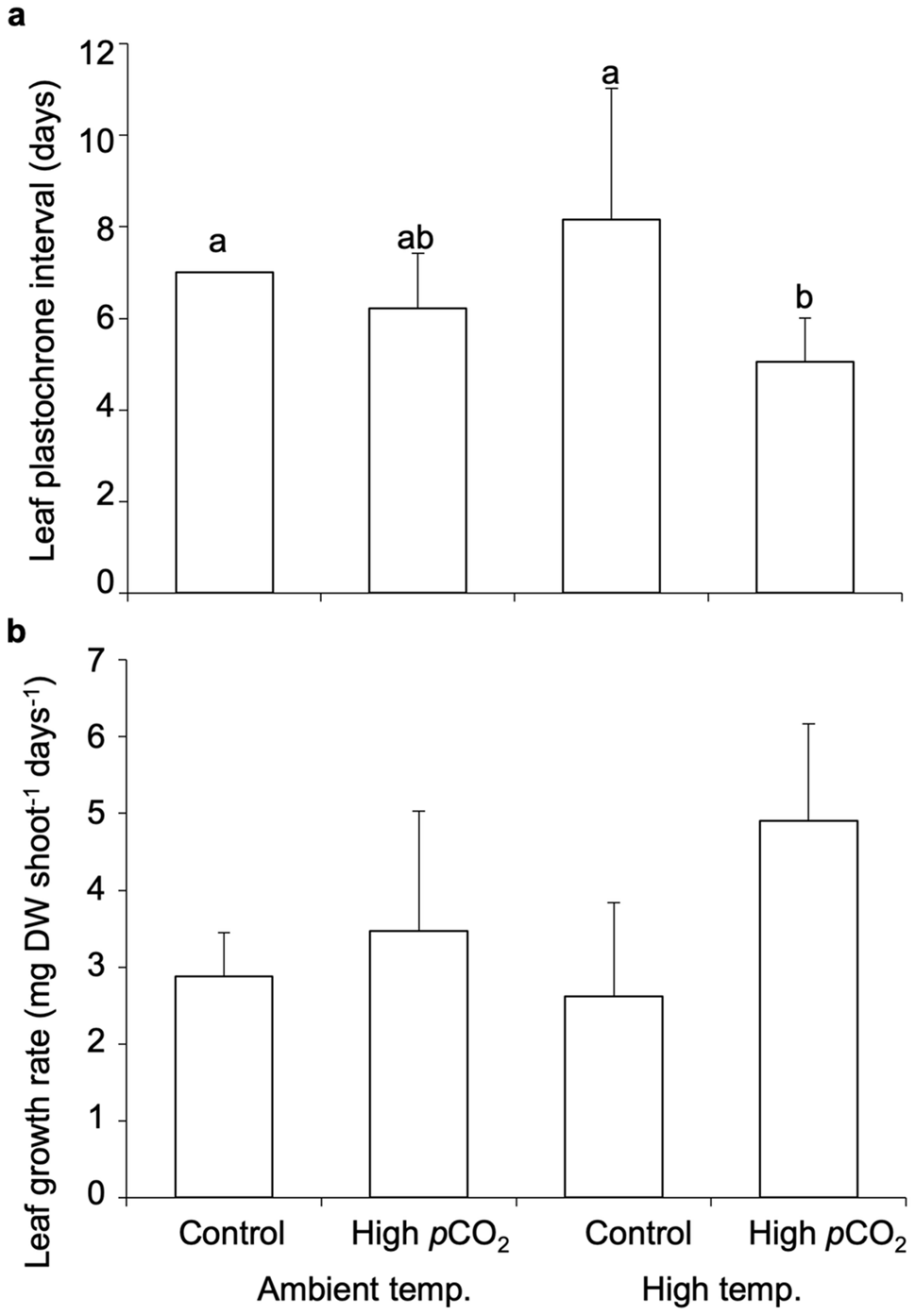
Effect of high temperature (+ 3 °C than ambient) and high *p*CO_2_ (1000 µatm) on the plasctochrone interval and growth rate of seagrass *Thalassia hemprichii*. (**a**) leaf plastochrone interval (P_L_); and (**b**) leaf growth rate. Values represent mean ± SD. n = 6. Different letters indicate statistically significant differences among treatment (Tukey’s HSD post-hoc test).

There was a significant interaction between *p*CO_2_ and temperature on leaf plastochrone interval (P_L_) of *Thalassia hemprichii* (GLM: F_(1,20)_ = 4.627, *p* = 0.044) and the shortest P_L_ (5.056 ± 0.952 days) was observed at high temperature and high *p*CO_2_ combined conditions (Tukey’s HSD post-hoc test: *p* < 0.05, Fig. 1a, Supplementary Table S1). High *p*CO_2_ significantly increased the leaf growth rate (two-way ANOVA: F_(1,20)_ = 8.472, *p* = 0.009), while there was no effects of temperature and interactive effects between the two factors (two-way ANOVA: *p* > 0.05, Fig. 1b, Supplementary Table S2).

### Seagrass photo-physiology

**Figure 2:**
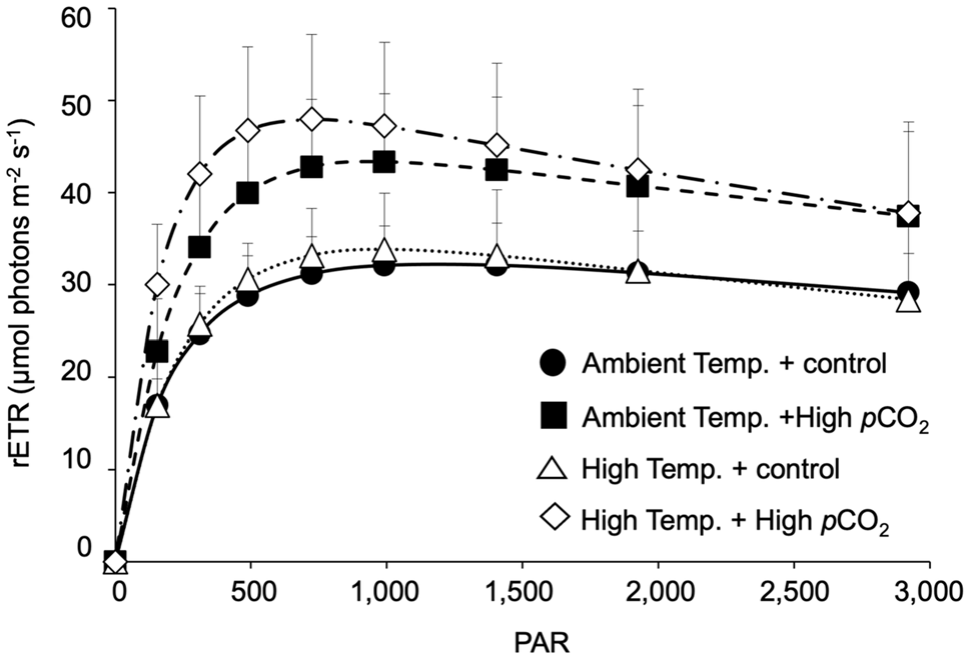
Effect of high temperature (+ 3 °C than ambient) and high *p*CO_2_ (1000 µatm) on rapid light curves (RLC) of seagrass *Thalassia hemprichii*. Values represent mean ± SD. n = 6.

High *p*CO_2_ significantly increased F_v_/F_m_ of *T. hemprichii* (two-way ANOVA: F_(1,20)_ = 4.954, *p* = 0.038), while there was no significant effect of high temperature and interaction between *p*CO_2_ and temperature (two-way ANOVA: *p* > 0.05, Table 1, Supplementary Table S3). The relative electron transport rate (rETR) value was highest for *T. hemprichii* cultured under the high temperature and high *p*CO_2_ combined condition (Fig. 2). Photo-physiological responses including α and rETR_max_ of *T. hemprichii* were significantly higher at high *p*CO_2_ (two-way ANOVA: α, F_(1,20)_ = 13.788, *p* = 0.001; rETR_max_, F_(1,20)_ = 18.286, *p* < 0.001) with no significant effect of high temperature and interaction between *p*CO_2_ and temperature (two-way ANOVA: *p* > 0.05). There was no effect of high *p*CO_2_ and temperature on β and E_k_ (two-way ANOVA: *p* > 0.05; Table 1, Supplementary Table S3).

**Table 1:** Effect of high temperature (+ 3 °C than ambient) and high *p*CO_2_ (1000 µatm) on photo-physiological parameters of seagrass *Thalassia hemprichii*. Values represent mean ± SD. n = 6.

Photo-physiological parameters	Ambient temperature		High temperature	
	Control	High *p*CO_2_	Control	High *p*CO_2_
F_v_/F_m_	0.727 ± 0.026	0.741 ± 0.022	0.670 ± 0.098	0.770 ± 0.060
α	0.16 ± 0.06	0.2 ± 0.06	0.15 ± 0.03	0.29 ± 0.07
β	0.003 ± 0.002	0.005 ± 0.004	0.005 ± 0.003	0.007 ± 0.003
rETR_max_	32.58 ± 4.49	43.57 ± 7.60	34.46 ± 6.02	48.20 ± 9.27
E_k_	239 ± 123	220 ± 44	241 ± 71	167 ± 33

### Seagrass carbon and nitrogen content

**Figure 3:**
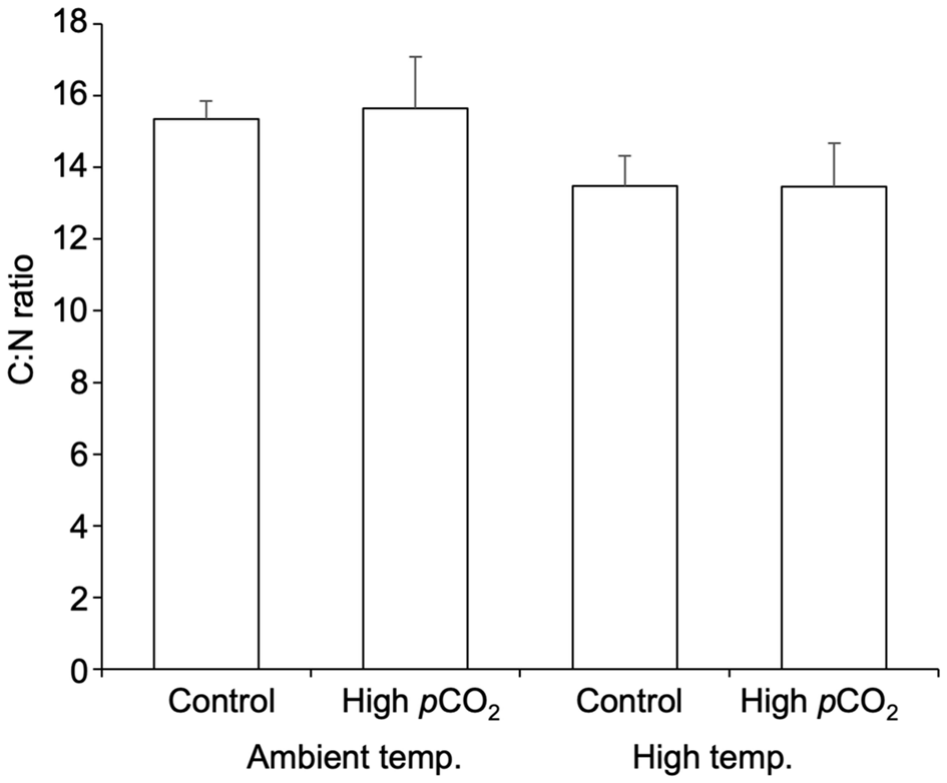
Effect of high temperature (+ 3 °C than ambient) and high *p*CO_2_ (1000 µatm) on the leaf C:N ratio of seagrass *Thalassia hemprichii*. Values represent mean ± SD. n = 6.

High temperature significantly decreased the above-ground leaf C:N ratio of *T. hemprichii* (two-way ANOVA: F_(1,20)_ = 21.756, *p* < 0.001), while there was no significant effect of high *p*CO_2_ and interaction between *p*CO_2_ and temperature (two-way ANOVA: *p* > 0.05, Fig. 3, Supplementary Table S4). Decreased C:N ratio at high temperature was caused by the significant increase of leaf nitrogen content, while leaf carbon content was not affected by both high temperature and high *p*CO_2_ (Supplementary Fig. S1a,c, Supplementary Table S4). The carbon content of below-ground part significantly decreased by high temperature, while there was no significant effect of *p*CO_2_ or interaction between temperature and *p*CO_2_ (Supplementary Fig. S1b, Supplementary Table S4). The nitrogen content of the below-ground part was significantly lower at high *p*CO_2_, with no significant effect of high temperature or interaction between the two factors (Supplementary Fig. S1d, Supplementary Table S4).

### Sea urchin feeding and fecal production rates

**Figure 4:**
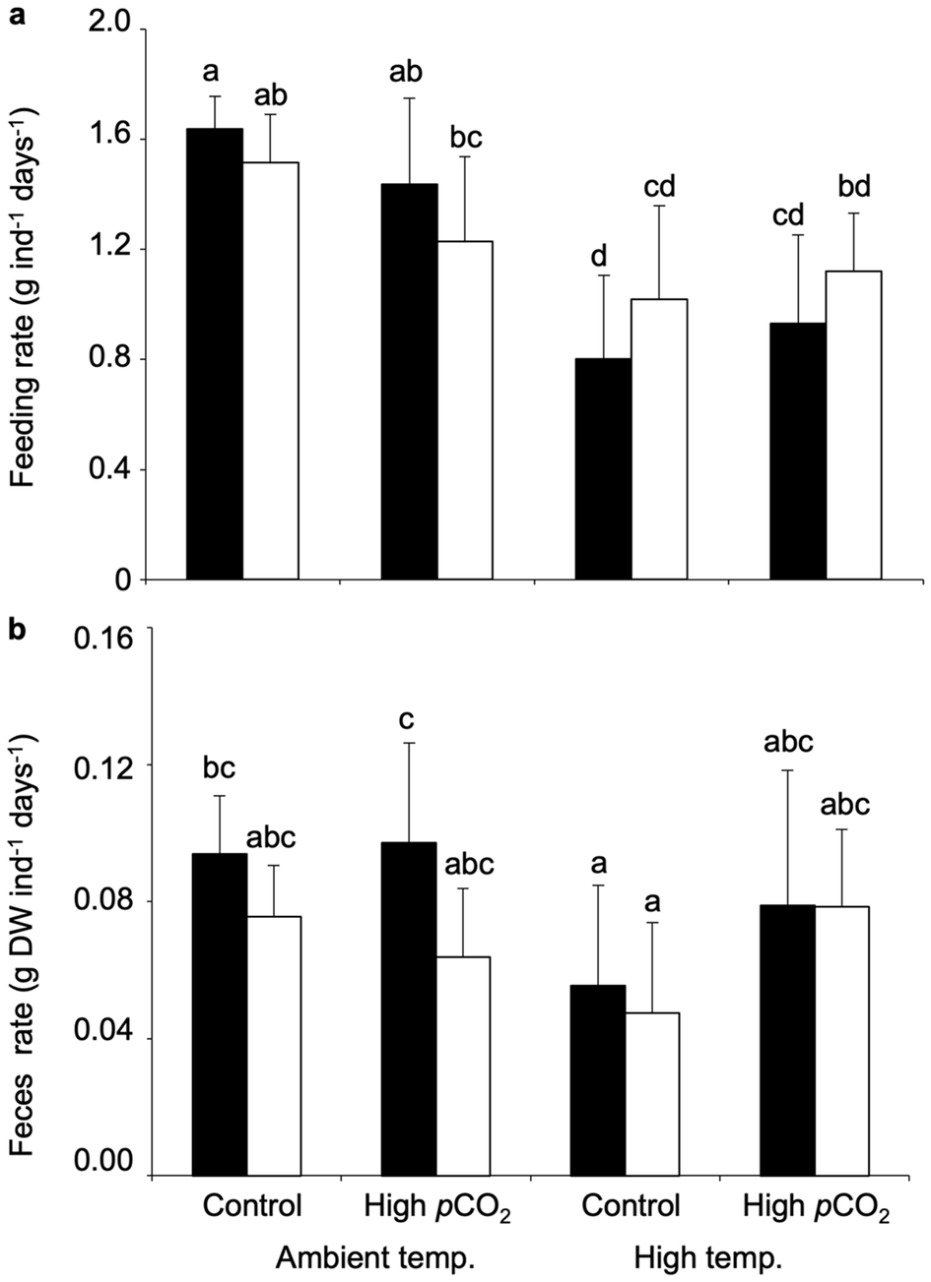
Effect of high temperature (+ 3 °C than ambient) and high *p*CO_2_ (1000 µatm) on feeding and fecal production rate of sea urchin *Tripneustes gratilla*. (**a**) Feeding rate and (**b**) fecal production rate of *T. gratilla* fed with experimental (black) and control (white) seagrass. Values represent mean ± SD. Ambient temperature and Control *p*CO_2_ (experimental seagrass: n = 10, control seagrass: n = 9), Ambient temperature and High *p*CO_2_ (experimental seagrass: n = 9, control leaves: n = 9), High temperature and Control *p*CO_2_ (experimental seagrass: n = 10, control seagrass: n = 8), High temperature and High *p*CO_2_ (experimental seagrass: n = 10, control seagrass: n = 8). Different letters indicate statistically significant differences among conditions (Tukey’s HSD post-hoc test).

The feeding rate of sea urchin *T. gratilla* showed significant interactive effects between *p*CO_2_ and temperature (GLM: F_(1,68)_ = 6.428, *p* = 0.014, Supplementary Table S5), and between the seagrass sources and temperature (GLM: F_(1,66)_ = 8.114, *p* = 0.006, Supplementary Table S5). *T. gratilla* showed the lowest feeding rate when cultured under high temperature and fed with seagrass cultured under high temperature conditions (Fig. 4a, Supplementary Table S5). Fecal production rate of *T. gratilla* showed significant interactive effects between *p*CO_2_ and temperature (three-way ANOVA: F_(1,65)_ = 6.460, *p* = 0.013, Fig. 4b, Supplementary Table S6). Additionally, seagrass treatments significantly affected the fecal production rate (three-way ANOVA: F_(1,65)_ = 6.171, *p* = 0.016; Fig. 4b, Supplementary Table S6).

Absorption efficiencies of carbon and nitrogen showed significant interaction between *p*CO_2_ and temperature, while there was no significant effect of seagrass treatments (Supplementary Fig. S2a, b, Supplementary Table S7).

### Sea urchin respiration and ammonium (NH_4_^+^) excretion rate

**Figure 5:**
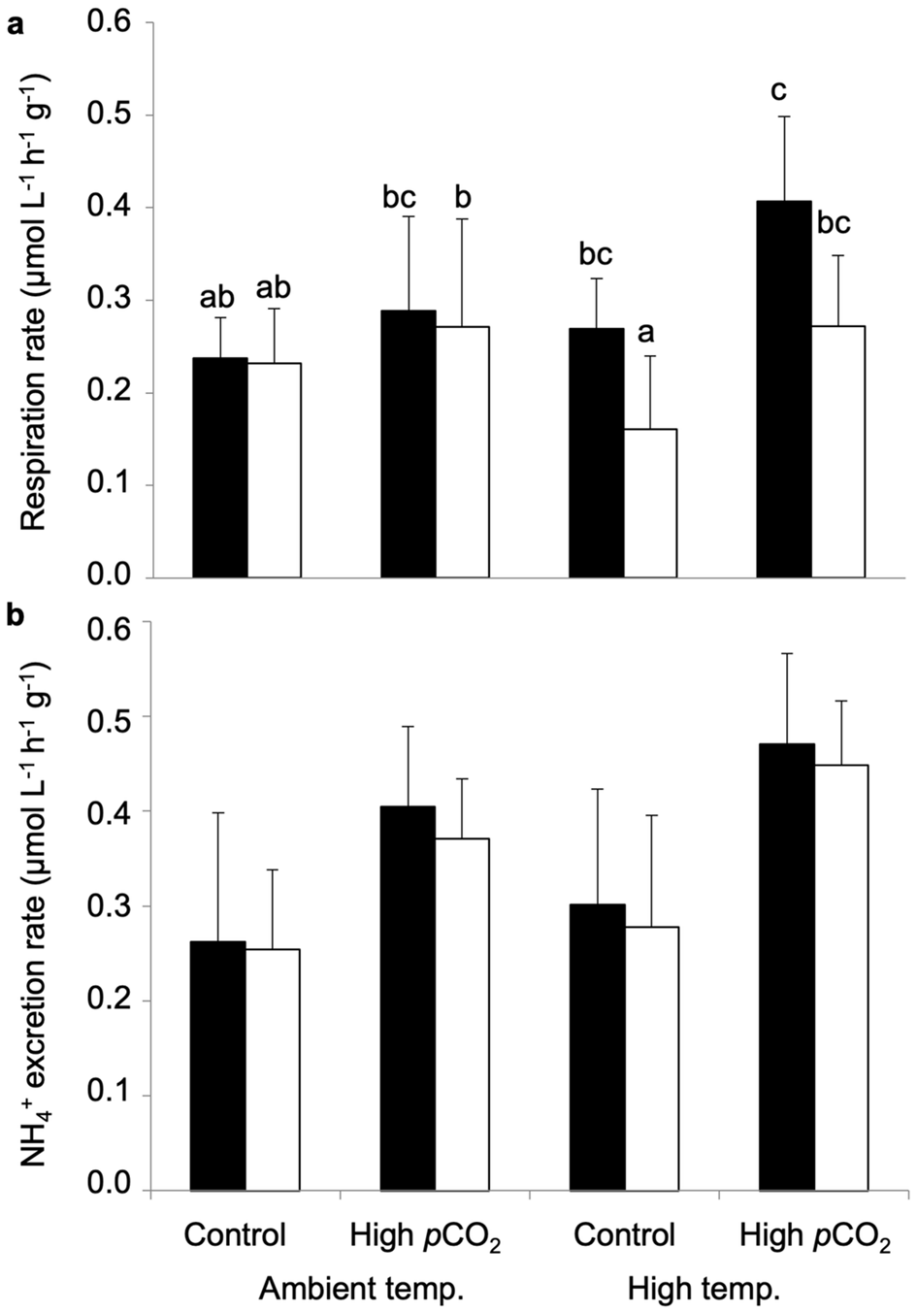
Effect of high temperature (+ 3 °C than ambient) and high *p*CO_2_ (1000 µatm) on respiration and ammonium excretion rates of sea urchin *Tripneustes gratilla*. (**a**) Respiration rate and (**b**) ammonium (NH_4_^+^) excretion rate of *T. gratilla* fed with experimental (black) and control (white) seagrass. Values represent mean ± SD. Ambient temperature and Control *p*CO_2_ (experimental seagrass: n = 9, control seagrass: n = 9), Ambient temperature and High *p*CO_2_ (experimental seagrass: n = 9, control seagrass: n = 9), High temperature and Control *p*CO_2_ (experimental seagrass: n = 9, control seagrass: n = 8), High temperature and High *p*CO_2_ (experimental seagrass: n = 8, control seagrass: n = 8). Different letters indicate statistically significant among conditions (Tukey’s HSD post-hoc test).

There was an interaction between *p*CO_2_ and temperature on the respiration rate of *T. gratilla* (three-way ANOVA: F_(1,61)_ = 4.655, *p* = 0.035; Fig. 5a and Supplementary Table S8). Additionally, respiration rate of *T. gratilla* was also interactively affected by the seagrass source and temperature (three-way ANOVA: F_(1,61)_ = 8.294, *p* = 0.005; Fig. 5a and Supplementary Table S8). Ammonium (NH_4_^+^) excretion rate of *T. gratilla* was significantly higher at high *p*CO_2_ (three-way ANOVA: F_(1,61)_ = 37.880, *p* < 0.001; Fig. 5b and Supplementary Table S8) and high temperature (three-way ANOVA: F_(1,61)_ = 4.673, *p* = 0.035; Fig. 5b and Supplementary Table S8), while there was no effect of seagrass treatments or interaction among *p*CO_2_, temperature, and seagrass treatments (three-way ANOVA: *p* > 0.05, Supplementary Table S8).

## Discussion

Increased seawater *p*CO_2_ was found to enhance the productivity of seagrass *T. hemprichii*. Meanwhile, the feeding rate of the tropical sea urchin *T. gratilla* on *T. hemprichii* significantly decreased particularly at high temperatures, suggesting a decrease in grazing pressure by this keystone herbivore in tropical seagrass meadows under ocean warming*.* These results indicate that climate change has the potential to cause a shift in the state of tropical seagrass meadows by changing both the bottom-up and top-down control.

Though increasing temperature can either positively or negatively affect seagrasses, photo-physiology and growth rate of *T. hemprichii* did not change at the high temperature condition. The optimum temperature for *T. hemprichii* at Santiago Islands, Philippines (annual temperature range of 24–33 °C) was reported to be around 27 °C^26^. Taking into account that the annual temperature range at the present site in Okinawa Island ranges from 20 to 30 °C^27^ and that the high temperature conditions did not show positive effects, it can be considered that the present high temperature condition (32.5 °C) was close to their upper thermal window range. Additionally, future temperature conditions may exceed the upper threshold of seagrass species that inhabit places with higher annual temperature including low latitude tropic regions. Consequently, although future increases of seawater temperature by 3 °C may not negatively impact *T. hemprichii* at Okinawa Island, further increases of temperature under ocean warming scenario may become deleterious to the seagrass.

Similar to prior studies on seagrasses^14–17^, ocean acidification enhanced photosynthetic rate parameters and increased the productivity of *T. hemprichii*. Additionally, rETR_max_ was also found to be enhanced under high *p*CO_2_ condition, following the finding that Rubisco activity of most seagrasses including *T. hemprichii* is undersaturated at the present *p*CO_2_ condition^28^. Therefore, it is predictable that ocean acidification will enhance the productivity of seagrass worldwide in the future. Interestingly, the P_L_ of *T. hemprichii* was synergistically enhanced by high *p*CO_2_ and temperature, and rETR although not significant, showed the highest value at the combined condition. Synergistic response to high *p*CO_2_ and temperature was also reported for the temperate seagrass *Zostera noltii* where high *p*CO_2_ tends to ameliorate the negative effects of high temperature on seagrass productivity^18^. One of the potential explanations is that high *p*CO_2_ condition increases the Rubisco activity and electron transport capacity inducing shifts of the optimum temperature to a higher temperature as suggested for terrestrial C_3_ plants^29^. Further studies are needed in order to evaluate those synergistic effects of temperature and *p*CO_2_ on seagrass.

In contrast to our hypothesis that sea urchin grazing will be enhanced by ocean warming and reduced by ocean acidification, here we found that high temperature reduced the feeding rate of *T. gratilla* while ocean acidification had no effect. Temperature rise is generally known to increase the feeding rate of herbivores due to the increase in their metabolic demands^20,30^. However, the respiration rate of *T. gratilla* did not change at the present high temperature condition (32.5 °C). Additionally, although increased temperature decreased the leaf C:N ratio, the feeding rate of *T. gratilla* fed both control and experimental seagrass decreased, suggesting direct negative effects of temperature on sea urchin physiology rather than indirect effects through seagrass nutrition. As such, it appears that *T. gratilla* populations in Okinawa are already living near their upper thermal limit and ocean warming will negatively affect *T. gratilla*, resulting in a decrease of grazing pressure on the seagrass.

Previous studies suggested that ocean acidification will decrease sea urchins feeding ability, because the exoskeletal structures including the feeding apparatus of sea urchins become more fragile when reared under high *p*CO_2_ condition^22^. Additionally, high *p*CO_2_ condition has been suggested to increase the C:N ratio of plants, although the effects of ocean acidification on seagrasses were not found to be uniform for both C:N ratio (increase^16,29,31^ or no change^19,20^) and for phenolic level (no change^19^ or decrease^23^). Here both feeding rate and fecal production rate of *T. gratilla* as well as C:N ratio of *T. hemprichii* were not affected by high *p*CO_2_, however both respiration and NH_4_^+^ excretion rates of *T. gratilla* were found to increase. Although most previous studies have reported no clear effect of high *p*CO_2_ on sea urchin respiration^21,32^, increased NH_4_^+^ excretion rate was also found in the sea urchin *Strongylocentrotus drobachiensis*^33^ and bivalves such as *Mytilus edulis,* which was suggested to indicate an increase of protein catabolism due to high *p*CO_2_^34^. These results suggest that although ocean acidification may not affect the grazing pressure of the sea urchin, it may affect the physiology of the sea urchin. Additionally, in terms of energy budget, it can be infered that increased respiration and NH_4_^+^ excretion rates with no significant change in feeding rate at high *p*CO_2_ could decrease the amount of energy available for growth and reproduction of the sea urchin. Indeed some previous studies have indicated that exposure of sea urchin to high *p*CO_2_ resulted in a decrease of feeding rate and delay in gonad development^21^, or increased respiration and reduced gonadosomatic index particularly in female sea urchins^35,36^. Furthermore, a decrease in the available energy for *T. gratilla* could be particularly significant under the combination of ocean warming and acidification, considering the significant decrease in feeding rate with the synergistic increase in respiration rate under high temperature and *p*CO_2_ environment, especially when *T. gratilla* was fed with experimental seagrass.

The present study demonstrated that ocean warming and acidification can show different direct impacts on plants and herbivores. Productivity of the seagrass was found to increase with ocean acidification, while grazing pressure by the tropical sea urchin will decrease under climate change, which can result in modification of plant–herbivore interactions. Plant–herbivore interactions have been suggested to structure tropical seagrass meadows, and ecosystem services by the tropical seagrass ecosystem, such as carbon sequestration and nutrient up-take, are maximized under the balanced system that support both seagrass and herbivore diversity^37,38^. Therefore, any alteration in the top-down control by herbivores such as sea urchins and the bottom-up control by the seagrass due to climate change can potentially result in a shift in the equilibrium regulating seagrass ecosystems leading to change in ecosystem functions and services of tropical seagrass meadow. Moreover, these effects can also potentially be strengthened further by concomitant stressors such as coastal development, eutrophication and overfishing.

## Methods

### Study species

Seagrass *Thalassia hemprichii* (Ehrenberg) Ascherson, 1871 shoots and sediment were collected at Bise (N 26° 42.548′, E 127° 52.740′) in Okinawa Island, Japan in May 2014. After being transported to the Sesoko Station, University of the Ryukyus, each seagrass shoot was carefully washed to remove epiphytes and sediment.

Eighty juvenile sea urchins *Tripneustes gratilla* (Linnaeus, 1758) of the same age were obtained from Okinawa Prefectural Sea Farming Center, Okinawa Island, Japan in February 2014. The sea urchins were transported to the Sesoko Station and cultured for 4 months in 4 tanks (157 L, n = 20 per tank) continuously supplied with filtered seawater (2 L min^−1^) and fed with *Undaria pinnatifida* every four days and were used as stock sea urchins for the following experiment.

### Experimental design

Two temperature conditions; ambient temperature and high temperature (+ 3 °C higher than ambient) and 2 *p*CO_2_ conditions; control (300–400 µatm) and high *p*CO_2_ (900–1000 µatm) were selected as present and year 2100 conditions according to the IPCC RCP 8.5 scenario^41^ (Table 2). Ambient seawater temperature fluctuated following field seawater by using flowing seawater pumped from 4–5 m depth in the front of the station. High temperature condition was controlled using heaters to be always 3 °C higher than the control. Seawater *p*CO_2_ was adjusted by bubbling seawater with air (control) or with a mixture of air and pure CO_2_ gas (high *p*CO_2_) controlled by mass flow controllers (Horiba Stec, SEC-E40, Japan). Both seagrass and sea urchins were acclimated for 40 days under the 2 temperatures and 2 seawater *p*CO_2_ full factorial design giving 4 experimental conditions before starting the measurements.

**Table 2:** Seawater carbonate chemistry during seagrass *Thalassia hemprichii* and sea urchin *Tripneustes gratilla* culture. Seawater *p*CO_2_, DIC and Ω_ar_ were calculated from the measured pH and total alkalinity (TA) using CO2SYS. Values represent mean ± SD.

Condition		*p*CO_2_ (µatm)	pH (NBS scale)	Temperature (°C)	Salinity	TA (µmol/kg)	DIC (µmol/kg)	Ωar
***T. hemprichii***								
Ambient Temp	Control	304 ± 42	8.27 ± 0.05	29.4 ± 1.6	34.2 ± 0.1	2231 ± 14	1848 ± 32	4.29 ± 0.38
	High *p*CO_2_	988 ± 219	7.85 ± 0.09	29.3 ± 1.6	34.2 ± 0.1	2232 ± 13	2075 ± 33	2.03 ± 0.39
High Temp	Control	337 ± 45	8.24 ± 0.05	32.4 ± 1.6	34.2 ± 0.1	2231 ± 13	1844 ± 30	4.42 ± 0.42
	High *p*CO_2_	930 ± 166	7.88 ± 0.07	32.4 ± 1.6	34.2 ± 0.1	2233 ± 13	2048 ± 30	2.34 ± 0.35
***T. gratilla***								
Ambient Temp	Control	375 ± 31	8.2 ± 0.03	28.7 ± 0.9	34.2 ± 0.2	2239 ± 10	1908 ± 19	3.74 ± 0.16
	High *p*CO_2_	895 ± 135	7.89 ± 0.06	28.7 ± 0.9	34.2 ± 0.2	2242 ± 12	2074 ± 21	2.11 ± 0.30
High Temp	Control	390 ± 31	8.19 ± 0.03	31.8 ± 0.8	34.2 ± 0.2	2239 ± 11	1890 ± 20	4.01 ± 1.60
	High *p*CO_2_	925 ± 137	7.88 ± 0.06	31.8 ± 0.7	34.2 ± 0.2	2240 ± 9	2059 ± 24	2.3 ± 0.28

Just after collection, the *T. hemprichii* were cut into one apical shoot with two rhizome internodes and roots, and 48 shoots were planted in each of 24 aquaria (12 L) containing 5 cm sediment thickness to mimic the density of *T. hemprichii* at the Bise site. Six aquaria were used as replicates for each of the 4 experimental conditions. The 4 experimental seawater conditions were continuously supplied (0.5 L min^−1^) to each of the 6 aquaria, and *T. hemprichii* were cultured for 40 days under natural sunlight until conducting the following measurements.

For the sea urchin, 40 individuals (3–4 cm diameter) were randomly selected from the stock and put individually in 40 containers (900 mL) with a mesh cage cylindrical lining inside each container. Replicate 10 containers received the 4 experimental seawater conditions (0.1 L min^−1^) and *T. gratilla* were cultured for 40 days in the laboratory under 12:12 h photoperiod artificial light (100 µmol photons m^−2^ s^−1^) controlled by 2 metal-halide lamps (W039-006P, Iwasaki, Japan). Sea urchins were fed with *Undaria pinnatifida* during the acclimation about once every 4 days.

During the seagrass and sea urchin culture, seawater pH (NBS scale), temperature, and salinity of each aquarium and containers were measured (14:00–15:00 h) using a multiparameter portable meter (WTW Multi 3420, Germany) connected with a temperature-compensated pH electrode (SenTix 940) and conductivity electrode (TetraCon 925). For total alkalinity (TA), seawater samples were taken every 2–3 days and measured using an auto-burrete titrator (Kimoto, ATT-05, Japan). Seawater *p*CO_2_ and Ω_aragonite_ were calculated based on pH, temperature, salinity, and TA data using CO2SYS ver. 2.1 program^39^ with K1 and K2 dissociation constants from Mehrbach recalculated by Dickson and Millero^40^ (Table 2).

### Seagrass leaf growth

The leaf growth of *T. hemprichii* was measured by the leaf plastochrone interval (P_L_) method^41^. After all the following sea urchin feeding experiments were finished, one apical seagrass shoot was chosen randomly from each of the 24 aquaria and punched using a needle at 1 cm from the lower part of the bundle sheath. The punched shoots were replanted into the aquarium and cultured for a further 14 days under the 4 experimental conditions. Thereafter, all 24 punched seagrass shoots were recollected, and P_L_ was calculated by dividing the number of days since marking (14 days) with the number of new leaves (unmarked leaves higher than the punch mark). Leaf growth (mg dry wt shoot^−1^ day^−1^) was calculated by dividing the dry weight measured using an electronic balance (HR-200, A&D, Japan) of the youngest mature leaf (the third leaf) dried (60 °C) for 7 days by the leaf P_L_.

### Seagrass photo-physiological responses

The photo-physiological responses of seagrass were measured using pulse amplitude modulated (PAM) fluorometry (Diving PAM, Walz, Germany) after the 40 days of being cultured. One apical shoot per aquarium was chosen randomly and placed in a clear container (8 L) with seawater equilibrated to the experimental condition it was previously reared at. After 15 min dark adaptation, saturation pulse (0.8 s) was applied to determine the maximum dark-adapted quantum yield of Φ_PSII_ (F_v_/F_m_) measured at the third fully developed leaf. Rapid light curve (RLC) was generated from relative electron transport rate (rETR) using 8 consecutive light levels of 155, 312, 488, 724, 992, 1406, 1926, and 2922 µmol photons m^−2^ s^−1^ applied every 10 s intervals. Derived RLC photosynthetic parameters including α (photosynthetic efficiency; the initial slope of the RLC before the saturation occurred), β (slope of the RLC when the photoinhibition occurred), maximum relative electron transport rate (rETR_max_), and E_k_ (minimum saturating irradiance) were calculated according to Platt et al.^42^, fitted using the Port method in the R Phytotools package^43^.

### Seagrass carbon and nitrogen content

Two shoots of seagrass that were not used for the above experiments were taken from each aquarium after the 40 days of culture. Epiphytes were scraped off of the seagrass leaves, and then they were divided into the above-ground part (leaves) and below-ground part (rhizomes and roots). Thereafter, all samples were dried (60 °C) for 7 days and the above- and below-ground parts of each of the two shoots were ground with a mortar and pestle into a homogenized fine powder. Ten mg of powder was weighed using an electronic balance (HR-202i, Japan) from each sample, and the carbon and nitrogen were measured the using CN analyzer (Sumigraph NC-22A, Japan).

### Sea urchin feeding and fecal production rate

To evaluate the sea urchins and seagrass interactive effects, feeding and fecal production rate of the sea urchins fed with the 2 seagrass treatments (experimental and control seagrass) were measured. All sea urchins were starved for 5 days after 35 days acclimation under the 4 experimental conditions. After starvation and taking all feces from each container, sea urchins in each of the 4 experimental conditions were fed with seagrass leaves that were cultured for 40 days under the same conditions as the sea urchins were cultured (experimental seagrass). Seagrass leaves (3.5 g, blot dried) were added to each container with the sea urchins. After 2 days, all the remnant leaves were collected, blotted dry, and weighed to calculate the feeding rate (g leaves ind^−1^ day^−1^). Additionally, all feces were collected from each container by filtering the seawater using pre-combusted (550 °C, 4 h) and pre-weighed fiberglass filter (Whatman GF/C). After removing all small remnant leaves using tweezers, each filter was dried at 60 °C until constant weight. The fecal production rate was calculated by subtracting the weight of filter containing feces with the filter weight (g dry feces ind^−1^ day^−1^). Additionally, to evaluate the carbon and nitrogen absorption efficiency, the dried feces were ground into a powder, and ten mg samples were weighed and fecal carbon and nitrogen content were measured with CN analyzer (Sumigraph NC-22A, Japan). Absorption efficiencies of carbon and nitrogen by sea urchin were calculated by the following formula:

$${\text{Absorption}}\,\,{\text{efficiency}}~\,(\% ) = ~\frac{{~{\text{element}}_{{{\text{leaves}}}} - {\text{element}}_{{{\text{feces}}}} }}{{{\text{element}}_{{{\text{leaves}}}} }}~ \times 100.$$

After the feeding experiment of experimental seagrass and the following respiration and ammonium excretion measurements detailed below, the same sea urchins were starved again for another 5 days. Thereafter all sea urchins were fed with the seagrass leaves cultured under the control condition (control seagrass). Two days later, the same procedure as above was repeated to measure the feeding and fecal production rate.

### Sea urchin respiration and ammonium (NH_4_^+^) excretion rate

Respiration and ammonium (NH_4_^+^) excretion rates of the sea urchins were measured just after the experimental and control seagrass feeding experiments, respectively. The next day after the feeding experiment, sea urchins were placed individually in 450 mL glass containers with a magnetic stirrer. After 24 h acclimation in continuously flowing experimental seawater, each glass container was closed tightly without headspace, and oxygen concentrations were measured 3 times at 0, 30, 60 min using FIBOX fiberoptic oxygen meter (Presens GmbH, Germany). Sea urchin respiration rate (µmol O_2_ L^−1^ h^−1^ g^−1^) was calculated by dividing the oxygen concentration change with seawater volume, incubation time, and wet weight (HR-200, A&D, Japan) of the sea urchin.

Concurrently with the respiration measurement, the ammonium (NH_4_^+^) excretion rate was measured by sampling seawater (1 mL) just before closing and just after opening each glass container containing sea urchins. Working reagent (250 µL) which consisted of borate buffer, sodium sulfite, and orthophthaldialdehyde (OPA) solution was added to each sample and incubated (2 h) in the dark (following Holmes et al.^44^). The NH_4_^+^ amount was measured colorimetrically (360 nm, UV-1800, Shimadzu, Japan), and the ammonium excretion rate (nmol NH_4_^+^ h^−1^ g^−1^) was calculated from the change of NH_4_^+^ concentration between the end and initial concentration, divided by seawater volume and wet weight of the sea urchin. After the ammonium excretion measurement, the sea urchins were starved to conduct the control seagrass feeding experiment, and then the same procedure was conducted again. All experimental protocols were approved by the University of the Ryukyus, and experiments were performed in accordance with appropriate guidelines and regulations, and in compliance with ARRIVE guidelines.

### Statistical analysis

All statistical analyses were calculated in R version 4.0.1^45^ using RStudio version 1.3.959^46^. All the data were checked for normality with the Shapiro–Wilk test and homogeneity of variances with the Levene’s test. Seagrass leaf growth, photo-physiological parameters, carbon and nitrogen content, and leaf C:N ratio were analyzed using two-way ANOVA with *p*CO_2_ and temperature as fixed factors. Data were transformed to meet assumptions of normality such as F_v_/F_m_ (x^4^ transformed), β (square-root(x) transformed), and E_k_ (log10(x) transformed). Sea urchin fecal production, respiration (square-root(x) transformed), and ammonium (NH_4_^+^) excretion rate were analyzed using three-way ANOVA with *p*CO_2_, temperature, and leaf sources as fixed factors. Data were further analyzed using Tukey’s HSD post-hoc test when the result of ANOVA test showed a significant interaction between the factors.

Data of seagrass leaf plastochrone interval (P_L_) and sea urchin feeding rate was analyzed using Generalized Linear Model (GLM). Inverse Gaussian was used to analyzed P_L_ with *p*CO_2_, temperature and their interaction were used as model variables. Quasi-Poisson was used to analyze sea urchin feeding rate with *p*CO_2_, temperature, leaf sources, and their interactions were used as model variables. When the interaction between independent variables was found, multiple pairwise comparisons analysis (Tukey–Kramer test) were applied using the multcomp package^47^.

## Supplementary Information

Supplementary Information.
