# Supplementary material for: Ocean warming and acidification modify top-down and bottom-up control in a tropical seagrass ecosystem
Source: Sci Rep. 2021 Jun 30;11:13605. doi: 10.1038/s41598-021-92989-0 (PMC8245495; doi:10.1038/s41598-021-92989-0)
Supplement: Supplementary file 1 — Supplementary Information. [file 41598_2021_92989_MOESM1_ESM.docx]

**Ocean warming and acidification modify top-down and bottom-up control in a tropical seagrass ecosystem**

**Supplementary Information**

Vina Listiawati^1,2,3^ & Haruko Kurihara^1*^

^1^Faculty of Science, University of the Ryukyus, Okinawa 903-0123, Japan

^2^Master of Marine Science, Universitas Diponegoro, Semarang 50275, Indonesia

^3^Department of Biology Education, Universitas Muhammadiyah Surakarta, Surakarta 57162, Indonesia

Email address: harukoku@sci.u-ryukyu.ac.jp (H. Kurihara)

Supplement Figures

**

**

**Figure S1**. Effect of high temperature (+3 °C than ambient) and high *p*CO_2_ (1,000 µatm) on (**a)** carbon content of above-ground part, (**b**) carbon content of below-ground part, (**c**) nitrogen content of above-ground part, and (**d**) nitrogen content of below-ground part of *Thalassia hemprichii*. Values represent mean ± SD. n = 6.

**

**

**Figure S2.** Effect of high temperature (+3 °C than ambient) and high *p*CO_2_ (1,000 µatm) on absorption efficiency of (**a**) carbon, and (**b**) nitrogen of *Tripneustes gratilla* fed with experimental (black) and control (white) seagrass. Values represent mean ± SD. Ambient temperature and control *p*CO_2_ (experimental seagrass: n = 10, control seagrass: n = 9), Ambient temperature and High *p*CO_2_ (experimental seagrass: n = 9, control seagrass: n = 9), High temperature and Control *p*CO_2_ (experimental seagrass: n = 10, control seagrass: n = 8), High temperature and High *p*CO_2_ (experimental seagrass: n = 10, control seagrass: n = 8). Different letters indicate statistically significant differences between temperature, high *p*CO_2_, and leaf treatments following a significant interaction (Tukey’s HSD post-hoc test).

Supplement Tables

| **Effect** | **df** | **Deviance** | **Residual df** | **Residual Deviance** | **F** | ***p*** |
| --- | --- | --- | --- | --- | --- | --- |
| Null |  |  | 23 | 0.227 |  |  |
| *p*CO_2_ | 1 | 0.080 | 22 | 0.147 | 11.446 | **0.003** |
| Temperature | 1 | 0.000 | 21 | 0.147 | 0.000 | 1 |
| *p*CO_2_ x Temperature | 1 | 0.032 | 20 | 0.114 | 4.627 | **0.044** |

| Model: GLM (plastochrone interval ~ *p*CO_2_*temperature, family = Inverse Gaussian)  AIC = 83.432  Deviance explained = 49.66% |
| --- |

**Table S1.** Result of generalized linear model (GLM) comparing the effects of high temperature (+3 °C than ambient) and high *p*CO_2_ (1,000 µatm) on the leaf plastochrone interval (P­­_L_) of seagrass *T. hemprichii*

| **Variable** | **Treatments** | **df** | **MS** | **F** | ***p*** |
| --- | --- | --- | --- | --- | --- |
| Leaf growth | *p*CO_2_ | 1 | 12.419 | 8.472 | **0.009** |
|  | Temperature | 1 | 2.071 | 1.413 | 0.249 |
|  | *p*CO_2_ x Temperature | 1 | 4.365 | 2.978 | 0.100 |
|  | Residuals | 20 | 1.466 |  |  |

**Table S2**. Result of two-way ANOVA comparing the effects of high temperature (+3 °C than ambient) and high *p*CO_2_ (1,000 µatm) on the leaf growth of seagrass *T. hemprichii*.

| **Variable** | **Treatments** | **df** | **MS** | **F** | ***p*** |
| --- | --- | --- | --- | --- | --- |
| F_v_/F_m_ | *p*CO_2_ | 1 | 0.039 | 4.954 | **0.038** |
|  | Temperature | 1 | 0.000 | 0.000 | 0.989 |
|  | *p*CO_2_ x Temperature | 1 | 0.021 | 2.665 | 0.118 |
|  | Residuals | 20 | 0.008 |  |  |
| α | *p*CO_2_ | 1 | 0.054 | 13.788 | **0.001** |
|  | Temperature | 1 | 0.009 | 2.422 | 0.135 |
|  | *p*CO_2_ x Temperature | 1 | 0.016 | 4.011 | 0.059 |
|  | Residuals | 20 | 0.004 |  |  |
| β | *p*CO_2_ | 1 | 0.000 | 1.799 | 0.195 |
|  | Temperature | 1 | 0.000 | 1.648 | 0.214 |
|  | *p*CO_2_ x Temperature | 1 | 0.000 | 0.005 | 0.945 |
|  | Residuals | 20 | 0.000 |  |  |
| rETR_max_ | *p*CO_2_ | 1 | 917.891 | 18.286 | **0.000** |
|  | Temperature | 1 | 63.416 | 1.263 | 0.274 |
|  | *p*CO_2_ x Temperature | 1 | 11.390 | 0.227 | 0.639 |
|  | Residuals | 20 | 50.197 |  |  |
| Ek | *p*CO_2_ | 1 | 0.031 | 1.506 | 0.234 |
|  | Temperature | 1 | 0.011 | 0.554 | 0.466 |
|  | *p*CO_2_ x Temperature | 1 | 0.034 | 1.648 | 0.214 |
|  | Residuals | 20 | 0.021 |  |  |

**Table S3.** Results of two-way ANOVA comparing the effects of high temperature (+3 °C than ambient) and high *p*CO_2_ (1,000 µatm) on the initial slope of rapid light curve (α), photoinhibition coefficient (β), maximum relative electron transport rate (rETR_max_), and minimum saturating irradiance (E_k_) of seagrass *T. hemprichii*.

| **Biomass** | **Variable** | **Treatments** | **df** | **MS** | **F** | ***p*** |
| --- | --- | --- | --- | --- | --- | --- |
| Above-ground  (leaf) | Carbon  (% DW) | *p*CO_2_ | 1 | 0.015 | 0.010 | 0.923 |
|  |  | Temperature | 1 | 1.895 | 1.178 | 0.291 |
|  |  | *p*CO_2_ x Temperature | 1 | 0.079 | 0.049 | 0.827 |
|  |  | Residuals | 20 | 1.608 |  |  |
|  | Nitrogen  (% DW) | *p*CO_2_ | 1 | 1.006 | 0.947 | 0.342 |
|  |  | Temperature | 1 | 12.441 | 11.708 | **0.003** |
|  |  | *p*CO_2_ x Temperature | 1 | 0.605 | 0.569 | 0.459 |
|  |  | Residuals | 20 | 1.063 |  |  |
|  | C:N | *p*CO_2_ | 1 | 0.120 | 0.107 | 0.747 |
|  |  | Temperature | 1 | 24.430 | 21.756 | **0.000** |
|  |  | *p*CO_2_ x Temperature | 1 | 0.163 | 0.145 | 0.707 |
|  |  | Residuals | 20 | 1.123 |  |  |
| Below-ground (rhizome and roots) | Carbon  (% DW) | *p*CO_2_ | 1 | 0.001 | 0.035 | 0.853 |
|  |  | Temperature | 1 | 0.326 | 9.859 | **0.005** |
|  |  | *p*CO_2_ x Temperature | 1 | 0.001 | 0.016 | 0.901 |
|  |  | Residuals | 20 | 0.033 |  |  |
|  | Nitrogen  (% DW) | *p*CO_2_ | 1 | 0.051 | 8.333 | **0.009** |
|  |  | Temperature | 1 | 0.020 | 3.205 | 0.089 |
|  |  | *p*CO_2_ x Temperature | 1 | 0.021 | 3.404 | 0.080 |
|  |  | Residuals | 20 | 0.006 |  |  |

**Table S4**. Results of two-way ANOVA comparing the effects of high temperature (+3 °C than ambient) and high *p*CO_2_ (1,000 µatm) on carbon and nitrogen content and C:N ratio of above-ground part (leaf), and carbon and nitrogen content of below-ground part (rhizome and roots) of seagrass *T. hemprichii*.

| **Effect** | **df** | **Deviance** | **Residual df** | **Residual Deviance** | **F** | ***p*** |  |
| --- | --- | --- | --- | --- | --- | --- | --- |
| Null |  |  | 72 | 9.582 |  |  |  |
| *p*CO_2_ | 1 | 0.085 | 71 | 9.497 | 1.220 | 0.273 |  |
| Temperature | 1 | 3.827 | 70 | 5.670 | 55.095 | **0.000** |  |
| Leaves | 1 | 0.003 | 69 | 5.667 | 0.040 | 0.842 |  |
| *p*CO_2_ x Temperature | 1 | 0.447 | 68 | 5.221 | 6.428 | **0.014** |  |
| *p*CO_2_ x Leaves | 1 | 0.009 | 67 | 5.211 | 0.136 | 0.714 |  |
| Temperature x Leaves | 1 | 0.564 | 66 | 4.648 | 8.114 | **0.006** |  |
| *p*CO_2_ x Temperature x Leaves | 1 | 0.001 | 65 | 4.647 | 0.009 | 0.924 |  |
| Model: GLM (plastochrone interval ~ *p*CO_2_*temperature*leaves, family = Quasi-Poisson)  AIC = NA  Deviance explained = 51.50% | | | | | | | |

**Table S5.** Results of generalized linear model (GLM) comparing the effects of high temperature (+3 °C than ambient), high *p*CO_2_ (1,000 µatm) and seagrass treatments (experimental vs control) on the feeding rate of sea urchin *T. gratilla*.

| **Variable** | **Treatments** | **df** | **MS** | **F** | ***p*** |
| --- | --- | --- | --- | --- | --- |
| Fecal production | *p*CO_2_ | 1 | 0.002 | 2.988 | 0.089 |
|  | Temperature | 1 | 0.006 | 8.439 | **0.005** |
|  | Leaves | 1 | 0.004 | 6.171 | **0.016** |
|  | *p*CO_2_ x Temperature | 1 | 0.004 | 6.460 | **0.013** |
|  | *p*CO_2_ x Leaves | 1 | 0.000 | 0.091 | 0.764 |
|  | Temperature x Leaves | 1 | 0.002 | 3.033 | 0.086 |
|  | *p*CO_2_ x Temperature x Leaves | 1 | 0.001 | 0.833 | 0.365 |
|  | Residuals | 65 | 0.001 |  |  |

**Table S6.** Results of three-way ANOVA comparing the effects of high temperature (+3 °C than ambient), high *p*CO_2_ (1,000 µatm) and seagrass treatments (experimental vs control) on the fecal production rate of sea urchin *T. gratilla*.

| **Variable** | **Treatments** | **df** | **MS** | **F** | ***p*** |
| --- | --- | --- | --- | --- | --- |
| Absorption efficiency of carbon | *p*CO_2_ | 1 | 0.364 | 0.086 | 0.771 |
|  | Temperature | 1 | 58.981 | 13.860 | **0.000** |
|  | Leaves | 1 | 11.302 | 2.656 | 0.108 |
|  | *p*CO_2_ x Temperature | 1 | 25.491 | 5.990 | **0.017** |
|  | *p*CO_2_ x Leaves | 1 | 1.207 | 0.284 | 0.596 |
|  | Temperature x Leaves | 1 | 3.232 | 0.759 | 0.387 |
|  | *p*CO_2_ x Temperature x Leaves | 1 | 1.658 | 0.390 | 0.535 |
|  | Residuals | 65 | 4.255 |  |  |
| Absorption efficiency of nitrogen | *p*CO_2_ | 1 | 0.001 | 0.000 | 0.990 |
|  | Temperature | 1 | 314.216 | 39.254 | **0.000** |
|  | Leaves | 1 | 12.273 | 1.533 | 0.220 |
|  | *p*CO_2_ x Temperature | 1 | 55.276 | 6.905 | **0.011** |
|  | *p*CO_2_ x Leaves | 1 | 0.697 | 0.087 | 0.769 |
|  | Temperature x Leaves | 1 | 71.704 | 8.958 | **0.004** |
|  | *p*CO_2_ x Temperature x Leaves | 1 | 0.017 | 0.002 | 0.963 |
|  | Residuals | 65 | 8.005 |  |  |

**Table S7**. Results of three-way ANOVA comparing the effect of high temperature (+3 °C than ambient), high *p*CO_2_ (1,000 µatm) and seagrass treatments (experimental vs control) on the absorption efficiency of carbon and nitrogen of sea urchin *T. gratilla*.

| **Variable** | **Treatments** | **df** | **MS** | **F** | ***p*** |
| --- | --- | --- | --- | --- | --- |
| Respiration rate | *p*CO_2_ | 1 | 0.102 | 17.011 | **0.000** |
|  | Temperature | 1 | 0.004 | 0.633 | 0.429 |
|  | Leaves | 1 | 0.072 | 12.114 | **0.001** |
|  | *p*CO_2_ x Temperature | 1 | 0.028 | 4.655 | **0.035** |
|  | *p*CO_2_ x Leaves | 1 | 0.000 | 0.001 | 0.981 |
|  | Temperature x Leaves | 1 | 0.050 | 8.294 | **0.005** |
|  | *p*CO_2_ x Temperature x Leaves | 1 | 0.000 | 0.082 | 0.776 |
|  | Residuals | 61 | 0.006 |  |  |
| Ammonium (NH_4_^+^) excretion rate | *p*CO_2_ | 1 | 0.376 | 37.880 | **0.000** |
|  | Temperature | 1 | 0.046 | 4.673 | **0.035** |
|  | Leaves | 1 | 0.008 | 0.803 | 0.374 |
|  | *p*CO_2_ x Temperature | 1 | 0.007 | 0.702 | 0.405 |
|  | *p*CO_2_ x Leaves | 1 | 0.001 | 0.069 | 0.793 |
|  | Temperature x Leaves | 1 | 0.000 | 0.002 | 0.961 |
|  | *p*CO_2_ x Temperature x Leaves | 1 | 0.001 | 0.084 | 0.773 |
|  | Residuals | 61 | 0.010 |  |  |

**Table S8.** Result of three-way ANOVA comparing the effect high temperature (+3 °C than ambient), high *p*CO_2_ (1,000 µatm) and seagrass treatments (experimental vs control) on the respiration and ammonium (NH_4_^+^) excretion rates of sea urchin *T. gratilla*.
